# Minimally invasive liver surgery for perihilar and intrahepatic cholangiocarcinoma: systematic review and meta-analysis of comparative studies

**DOI:** 10.1007/s00464-025-11900-4

**Published:** 2025-09-10

**Authors:** Joey de Hondt, Maurice J. W. Zwart, Bas A. Uijterwijk, George L. Burchell, Burak Görgeç, Babs Zonderhuis, Geert Kazemier, Joris Erdmann, Marc G. Besselink, Rutger-Jan Swijnenburg

**Affiliations:** 1 Department of Surgery, Amsterdam UMC, Location University of Amsterdam, Amsterdam, the Netherlands; 2 Department of Surgery, Amsterdam UMC, Location Vrije Universiteit Amsterdam, Amsterdam, the Netherlands; 3 Cancer Center Amsterdam, Amsterdam, the Netherlands; 4 Department of Medical Informatics, Vrije Universiteit Amsterdam, Amsterdam, the Netherlands

**Keywords:** Cholangiocarcinoma, Minimally invasive, Hepatectomy, Robotic

## Abstract

**Background:**

The implementation of minimally invasive liver surgery (MILS) for perihilar (PHC) and intrahepatic cholangiocarcinoma (IHC) remains limited and a systematic review including only comparative studies of MILS versus the open approach is lacking. This systematic review and meta-analysis aimed to assess the safety and efficacy of minimally invasive surgery in patients with hilar and intrahepatic cholangiocarcinomas.

**Methods:**

Systematic review in the PubMed, Embase, and Cochrane databases for original studies comparing at least five patients undergoing MILS with open liver surgery for PHC and IHC. Meta-analysis included the primary outcomes of morbidity and mortality. Secondary outcomes included post-operative outcomes, recurrence, disease-free survival, and resection margins.

**Results:**

Overall, 37 comparative non-randomised studies with 4863 patients were included, of which 24% PHC and 76% IHC. In 21 studies, propensity score matching was performed. In total, 2106 laparoscopic, 75 robotic, and 2662 open procedures were analysed. The conversion rate was median 11.5% [IQR 10.0–12.5]. MILS probably resulted in reduced rates of major morbidity, 13.3% vs 18.8% (OR 0.75, 95%CI 0.62–0.90), mortality, 3.0% vs 4.5% (OR 0.69, 95%CI 0.49–0.97), and shorter hospital stay, 8.0 vs 10.9 days (MD -2.1, 95%CI -2.8 – -1.5). MILS resulted in higher rate of R0 resections in PSM cohort, 90.4% vs 81.4%, (OR 1.40, 95%CI 1.13–1.74) and better 3-year disease-free survival rate (49.9% vs 38.5%, HR_3-year_ 3.2, 95%CI 3.1–3.3). In the subgroup of 1180 patients in whom a hepatico-jejunostomy was performed (498 laparoscopic, 65 robotic, 617 open) MILS remained associated with reduced major morbidity, 20.9% vs 27.6% (OR 0.88, 95%CI 0.64–1.21) and resulted in better mortality, 4.2% vs 4.9% (OR 0.51, 95%CI 0.30–0.86), as compared to the open approach. Overall, the rate of biliary leakage was likely similar, 10.6% versus 11.7% (OR 0.83, 95%CI 0.52–0.77).

**Conclusion:**

This systematic review of non-randomised comparative studies suggests that MILS for PHC and IHC may result in a similar safety profile with benefits in patient recovery and oncological outcomes as compared to OLS. Prospective comparative studies, especially including robotic MILS, are warranted.

Cholangiocarcinoma originates from the cholangiocytes in the biliary system [1–3]. Cholangiocarcinoma can be located intrahepatically (IHC), perihilar (PHC), and in the distal common bile duct, all of which are uncommon, aggressive types of cancer with a poor prognosis [1]. The prognosis of cholangiocarcinoma is rather poor, with a median survival after resection of 31 to 37 months [1, 4–6]. Radical surgical resection is the only curative treatment, although the procedure can be challenging. Surgery for IHC and PHC can involve (extended) hemihepatectomy with lymphadenectomy, which is oftentimes combined with resection of the extrahepatic bile duct, necessitating a biliary reconstruction [1, 3, 7, 8]. The high levels of mortality and morbidity reflect the challenges faced operatively and post-operatively [7, 8]. Due to advancements in surgical techniques and post-operative care, there have been major improvements over time in terms of morbidity and survival [1, 8, 9].

Although minimally invasive liver surgery (MILS) could potentially improve recovery and wound complication rates due to the high complexity and fear of inferior oncological outcomes, MILS for cholangiocarcinoma is only slowly adopted [9–15]. MILS has therefore been limited to specific cases only [10, 11]. Moreover, the limited knowledge about the efficacy of MILS limits its further implementation. Earlier systematic reviews of non-comparative studies have suggested some benefits of MILS for short-term outcomes, but uncertainty for long-term outcomes, such as oncological effectiveness [12, 16–23]. Recent systematic reviews of comparative studies have shown similar results, although for specific tumour locations and with a limited study population [24–26]. A large systematic review including only comparative studies of MILS versus open liver surgery (OLS) for non-distal cholangiocarcinoma is lacking.

The aim of this systematic review is to summarise the current knowledge on the safety and effectiveness of MILS versus OLS for the treatment of selected patients with PHC and IHC.

## Materials and methods

### Research aims and outcomes

This systematic review with meta-analysis (CRD42017074398) is reported in line with the PRISMA 2020 guidelines and primarily aims to compare mortality and major morbidity after MILS versus OLS in selected patients with PHC and IHC. Secondary outcomes included operation time, blood loss, wound infection, and hospital stay. The other outcomes were defined in operative outcomes and oncological outcomes. Operative outcomes included readmission rate and the extent of resection. Oncological outcomes included recurrence, disease-free survival (DFS), resection margins, and number of lymph nodes resected. GRADE framework was used to assess certainty of evidence [68].

### Search strategy

For this systematic review the PubMed, Embase, and Cochrane databases were searched for publications involving minimally invasive surgery, including laparoscopic, robotic, hybrid, and hand-assisted laparoscopic surgery, in patients with PHC and IHC. All original comparative study designs published since inception up to 1st of June, 2024 were included. Cohorts smaller than five were excluded, because of possible high risk of biases due to an inflated effect size estimation with a small cohort size with too low power to detect confounding. The search strategy was developed according to a population, intervention, control format, in collaboration with a librarian (GB) (see Appendix 1).

### Data extraction and collected variables

First, title and abstracts were screened by two researchers (MZ & JdH). Conflicts were resolved in a consensus meeting. Second, full texts were screened for eligibility based on the inclusion and exclusion criteria. Risk of bias was assessed using the Newcastle Ottawa Scale (NOS); attainable score 1–9 [27]. Two reviewers independently assessed each study for risk of bias. Conflicts were resolved in a consensus meeting. Studies scoring ≥ 7 were considered low risk of bias, 4–6 moderate risk, and ≤ 3 high risk of bias [28]. Data retrieved from the included studies consisted of article details (year of publication, author, title, sample size, study type, -period, and -demographics), patient characteristics (sex, age, follow-up time, tumour size, location/type of tumour), operative specifics (operation time, blood loss, technical approach (hand-assisted, full laparoscopic, robotic, or hybrid), conversion, extent of resection, number of lymph nodes resected), and post-operative outcomes (90-day mortality, morbidity, resection margins, hospital stay, wound infection, hospital costs, recurrence, readmission, and disease-free survival). Extent of resection was grouped in either minor or major resections. Major resections were defined as a resection of three or more liver segments. MILS volume (per year) was calculated based on the months of inclusion and the number of patients included in the studies, preferably the number of patients eligible for inclusion were used over the number included.

### Statistical analysis

The IBM SPSS Statistics 28, Armonk, New York, United States, package was used for the statistical analysis. A meta-analysis was performed; statements were formulated whilst adhering to GRADE guidelines 26. [29] Statistical analyses were conducted with the assistance of Dr. S. van Dieren, a biostatistician and epidemiologist. The dichotomous results were presented by odds ratio (OR) with 95% confidence interval (CI). Continuous data were presented by mean difference (MD) with 95% CI. Heterogeneity amongst studies was estimated using the I^2^-test. When no significant heterogeneity (I^2^ < 40%) was estimated a fixed-effect model was used. Otherwise, a random-effect model was used. Time-to-event meta-analysis was used to analyse the DFS. Furthermore, funnel plots were performed to assess the publication bias. This form of bias was deemed minimal if a symmetrical funnel plot was calculated. Statistical significance was determined at P < 0.050. A further subgroup analysis of this meta-analysis was performed. Studies were analysed with respect to the following variables: type of tumour (subgroup analysis for intrahepatic or perihilar), type of minimally invasive treatment (subgroup analysis hand-assisted, robotic, or laparoscopic), and propensity score matched studies. Sensitivity analysis comprised the following variables: age of patients (meta-regression analyses), annual study volume (meta-regression analyses based on the study period and cases included), extent of resection, and biliodigestive anastomosis. A difference in the pooled effect size indicated an effect of the respective variable on the outcome. A further sensitivity analysis was performed by removing the largest studies and reviewing differences in the primary-, secondary-, and oncologic outcomes. Continuous data was extracted together with the standard deviation. The standard deviation was derived from the interquartile-range [IQR] or the range if it was itself not mentioned in the study [30, 31].

## Results

### Study selection

**Fig. 1:**
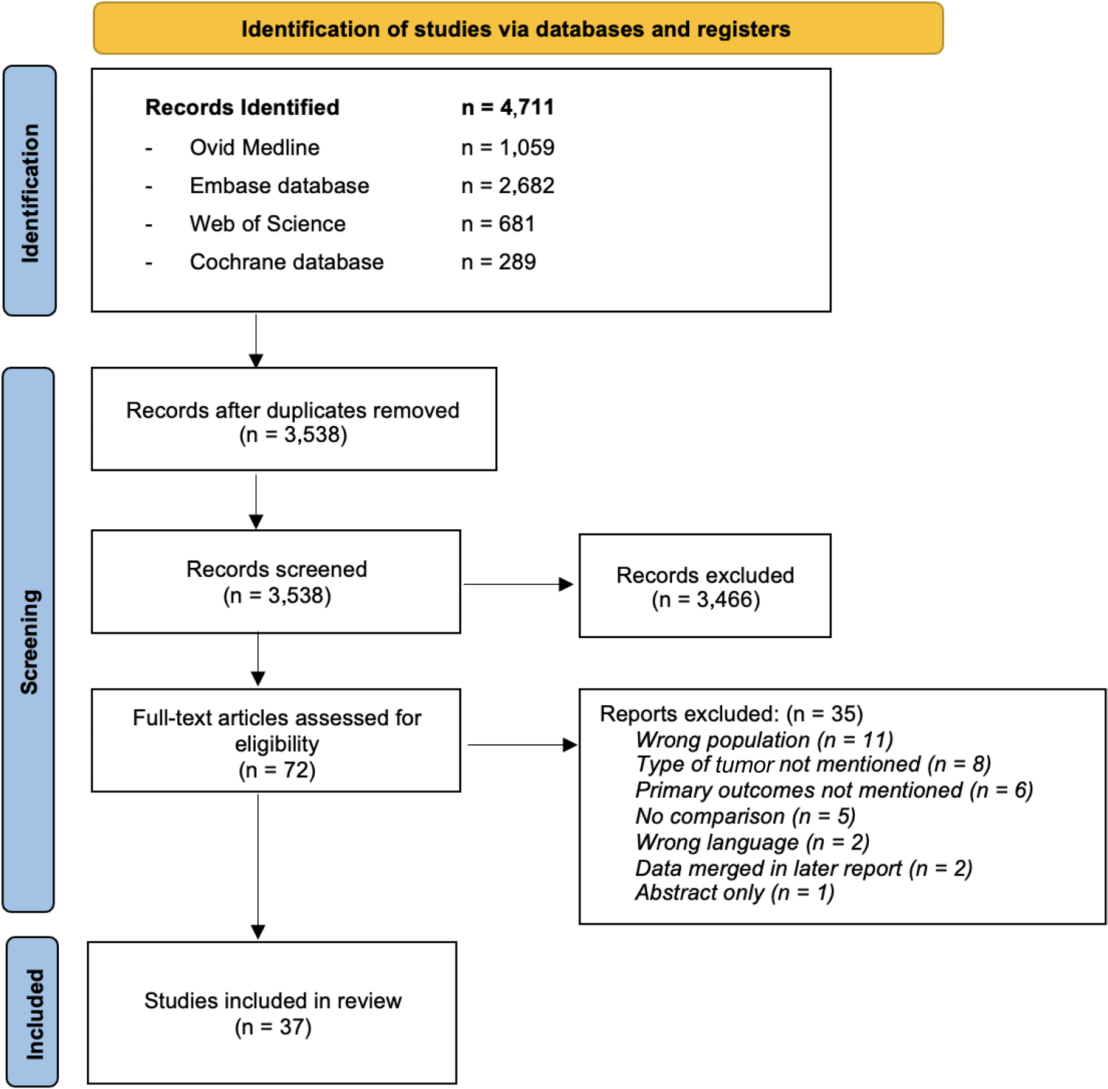
Flow diagram of included studies

The literature search yielded 4711 studies of which 3538 were unique. After screening titles and abstracts, the full text of 72 studies were screened, which resulted in the inclusion of 37 studies [16, 32–67]. Figure 1 shows the PRISMA flow diagram of the study selection.

### Baseline characteristics

#### Study characteristics

A total of 21 studies were propensity score matched cohorts [16, 32, 34, 36–38, 40, 42, 45, 48, 50, 53, 54], which included eight multicentre studies [18, 32, 34, 35, 50, 57, 61, 66], of which one national cancer database analysis[33], and two international database analysis [18, 50]. Except for one study [49], all studies were of a retrospective design. The study periods ranged from 2000 to 2023. Overall, 4863 patients were included, of which 2181 (45.0%) received MILS and 2662 (54.0%) received OLS. The conversion rate, reported by 30 studies, was 11.1% (range 0.0%─40.0%). The baseline characteristics of the included articles are summarised in Table 1. The funnel plot of study size and the primary outcomes was symmetrical, no evidence of publication bias was found for morbidity, major morbidity, nor mortality (see Appendix 2).

**Table 1:** Study characteristics

First author	Year	Country	Design	Approach	Sample Size		Period
					MILS	OLS	
Uy [36]	2015	South Korea	Cohort	Laparoscopic	11	26	2004 – 2012
Ratti [38]	2015	Italy	PSM	Laparoscopic	20	60	2004 – 2014
Lee [39]	2016	South Korea	Cohort	Laparoscopic	14	23	2010 – 2015
Wei [33]	2016	China	Cohort	Laparoscopic	12	20	2004 – 2016
Xu [44]	2016	China	Cohort	Robotic	10	32	2009 – 2012
Ratti [54]	2018	Italy	PSM	Laparoscopic	104	208	2004 – 2017
Cipriani [16]	2019	International	PSM	Laparoscopic	545	545	2007 – 2016
Zhang [47]	2019	China	Cohort	Laparoscopic	18	25	2015 – 2018
Zhu [53]	2019	China	PSM	Laparoscopic	18	36	2012 – 2017
Efanov [46]	2020	Russia	Cohort	Robotic	13	88	2014 – 2018
Haber [43]	2020	Germany	Cohort	Lap. hybrid	27	31	2015 – 2019
Kang [40]	2020	South Korea	PSM	Laparoscopic	24	24	2004 – 2015
Ratti [48]	2020	Italy	PSM	Laparoscopic	16	32	2004 – 2019
Ratti [42]	2020	England & Italy	PSM	Laparoscopic	104	104	2004 – 2017
Wu [41]	2020	China	Cohort	Laparoscopic	18	25	2010 – 2017
Chen [64]	2021	China	Cohort	Laparoscopic	23	42	2014 – 2020
Dou [63]	2021	China	Cohort	Laparoscopic	17	17	2017 – 2020
Hobeika [32]	2021	France	PSM	Laparoscopic	109	109	2000 – 2017
Ma [45]	2021	China	Cohort	Laparoscopic	40	78	2015 – 2020
Ratti [37]	2021	Italy	PSM	Laparoscopic	150	150	2004 – 2020
Salehi [34]	2021	USA	PSM	Laparoscopic	115	115	2010 – 2016
Brustia [50]	2022	International	PSM	Laparoscopic	89	89	2000 – 2018
Efanov [62]	2022	Russia	PSM	Robotic	17	33	2013 – 2021
He [56]	2022	China	PSM	Laparoscopic	16	32	2018 – 2022
Jinhuan [61]	2022	China	PSM	Laparoscopic	122	122	2011 – 2018
Ma [59]	2022	China	PSM	Laparoscopic	20	47	2017 – 2020
Shapera [49]	2022	USA	PSM	Robotic	15	19	2016 – 2021
Sucandy [51]	2022	USA	PSM	Robotic	10	16	2016 – 2021
Wang [52]	2022	China	Cohort	Laparoscopic	30	65	2011 – 2021
Huang [55]	2023	China	PSM	Robotic	10	20	2017 – 2022
Liu [60]	2023	China	Cohort	Laparoscopic	38	54	2017 – 2020
Pery [58]	2023	USA	Cohort	Laparoscopic	32	52	2016 – 2021
Qin [35]	2023	China	PSM	Laparoscopic	83	83	2013 – 2018
Sahakyan [57]	2023	International	PSM	Laparoscopic	50	50	2012 – 2019
Shen [67]	2023	China	PSM	Laparoscopic	70	35	2010 – 2021
Wang [66]	2023	China	PSM	Laparoscopic	141	141	2013 – 2019
Xiong [65]	2023	China	Cohort	Laparoscopic	34	30	2018 – 2020

*PSM* Propensity score matched,* Lap* Laparoscopic,* USA * United Stated of America

#### Patient characteristics

Median age was similar between MILS versus OLS, 63 years versus 63 years, respectively (MD − 0.400, 95%CI − 1.48 – 0.68, P = 0.305, I^2^ = 0.64). Tumour size was significantly smaller in patients undergoing MILS: 38.8 mm (SD 16.1) versus 51.4 mm (SD 13.0) (MD − 3.44, 95%CI −5.19 – − 1.68, P = 0.007, I^2^ = 0.85). Thirteen studies reported on patients with PHC (24.4%, 1185 patients) and 24 studies on IHC (75.6%, 3678 patients) [16, 32–34, 36–43, 49–54, 57–59, 61, 64, 67]. No comparative studies for minimally invasive- vs open combined liver resection and pancreatoduodenectomy. Nineteen studies performed biliodigestive anastomosis [33, 35, 41, 44–50, 53, 55–58, 60, 62, 65, 66], of which 12 were in a cohort focussing on perihilar cholangiocarcinoma [35, 44–48, 55, 56, 60, 62, 65, 66]. Some 34 studies reported the extent of resection. The rate of minor resections was lower in MILS compared to OLS: 36.7% versus 34.7% (OR 1.38, 95%CI 1.01–1.88 P = 0.041, I^2^ = 0.72). One study reported a hand-assisted or hybrid approach, and six studies reported a robotic approach (75 patients) [44, 46, 49, 51, 55, 62]. For more information, see Table 2.

**Table 2:** Patient characteristics included studies

First author	Tumour size (mean ± SD)		Tumour location	Gender male/female		Age (mean ± SD)	
	MILS	OLS		MILS	OLS	MILS	OLS
Uy [36]	4.2 ± 4.0	4.3 ± 3.2	Intrahepatic	9/2	18/8	67.0 ± 8.8	67.0 ± 21.8
Ratti [38]	3.9 ± 1.3	4.3 ± 1.2	Intrahepatic	12/8	34/26	59.0 ± 6.0	62.0 ± 5.0
Lee [39]	3.5 ± 1.1	4.0 ± 1.7	Intrahepatic	11/3	19/4	66.0 ± 7.8	59.0 ± 7.3
Wei [33]	N/A	N/A	Intrahepatic	12/0	N/A	60.5 ± 5.8	N/A
Xu [44]	N/A	N/A	Perihilar	8/2	20/12	54.0 ± 10.3	59.0 ± 20.2
Ratti [54]	3.9 ± 1.3	4.3 ± 1.2	Intrahepatic	64/40	120/84	59.0 ± 6.0	62.0 ± 5.0
Cipriani [16]	N/A	N/A	Intrahepatic & metastases	302/243	332/213	N/A	N/A
Zhang [47]	2.3 ± 0.8	2.8 ± 2.0	Perihilar	7/7	3/6	65.4 ± 8.9	65.4 ± 6.9
Zhu[53]	6.0 ± 1.5	6.0 ± 1.3	Intrahepatic	10/8	19/17	54.1 ± 16.6	55.6 ± 9.8
Efanov [46]	N/A	N/A	Perihilar	6/7	47/41	61.0 ± 7.5	61.0 ± 20.4
Haber [43]	N/A	N/A	Intrahepatic	13/14	18/13	69.0 ± 9.8	63.0 ± 12.3
Kang [40]	4.7 ± 3.3	4.1 ± 1.8	Intrahepatic	15/9	15/9	66.8 ± 9.7	68.1 ± 10.2
Ratti [48]	N/A	N/A	Perihilar	8/8	17/15	61.0 ± 8.3	63.0 ± 9.3
Ratti [42]	3.9 ± 1.7	4.1 ± 1.2	Intrahepatic	70/34	68/36	59.0 ± 5.0	61.0 ± 6.0
Wu [41]	N/A	N/A	Intrahepatic	12/6	10/15	65.3 ± 8.9	60.0 ± 6.7
Chen [64]	N/A	N/A	Intrahepatic	N/A	N/A	N/A	N/A
Dou [63]	N/A	N/A	Perihilar	N/A	N/A	N/A	N/A
Hobeika [32]	N/A	N/A	Intrahepatic	N/A	N/A	67.0 ± 8.9	61.0 ± 11.9
Ma [45]	2.5 ± 0.9	2.5 ± 1.0	Perihilar	16/4	38/9	61.9 ± 9.00	61.0 ± 8.30
Ratti [37]	5.3 ± 2.3	5.8 ± 1.2	Intrahepatic	92/58	86/64	61.0 ± 4.0	62.0 ± 7.0
Salehi [34]	4.2 ± 0.8	4.4 ± 1.0	Intrahepatic	42/73	37/78	65.7 ± 8.8	67.1 ± 7.5
Brustia [50]	4.7 ± 2.6	5.3 ± 3.7	Intrahepatic	52/37	51/38	65.2 ± 11.4	67.9 ± 9.0
Efanov [62]	N/A	N/A	Perihilar	N/A	N/A	N/A	N/A
He [56]	2.6 ± 1.1	2.7 ± 1.0	Perihilar	7/9	16/16	62.0 ± 3.0	62.5 ± 3.7
Jinhuan [61]	N/A	N/A	Intrahepatic	68/54	73/49	N/A	N/A
Shapera [49]	5.5 ± 2.8	5.5 ± 4.3	Intrahepatic	10/5	13/6	68.4 ± 12.1	60.7 ± 11.4
Sucandy [51]	6.0 ± 3.8	7.0 ± 4.0	Intrahepatic	N/A	N/A	61.0 ± 12.5	64.0 ± 12.1
Wang [52]	4.7 ± 0.4	5.7 ± 0.3	Intrahepatic	11/19	37/28	60.6 ± 9.4	61.7 ± 9.0
Huang [55]	2.3 ± 1.3	2.3 ± 0.7	Perihilar	7/3	12/8	66.0 ± 8.9	65.0 ± 14.1
Liu [60]	N/A	N/A	Perihilar	20/18	25/29	56.7 ± 12.3	64.2 ± 10.5
Pery [58]	4.0 ± 3.1	5.6 ± 2.7	Intrahepatic	13/19	21/31	65.0 ± 10.4	68.8 ± 7.4
Qin [35]	2.7 ± 1.1	2.8 ± 1.1	Perihilar	40/43	44/39	62.1 ± 9.4	62.7 ± 10.3
Sahakyan [57]	5.5 ± 3.2	5.8 ± 2.9	Intrahepatic	23/27	22/28	65.5 ± 9.2	65.4 ± 10.6
Shen [67]	4.9 ± 1.8	5.3 ± 3.3	Intrahepatic	35/35	17/18	66.0 ± 9.6	66.0 ± 10.4
Wang [66]	2.7 ± 1.2	2.8 ± 1.3	Perihilar	86/55	86/55	62.6 ± 9.2	62.1 ± 9.0
Xiong [65]	2.0 ± 0.4	2.0 ± 0.4	Perihilar	18/16	14/16	55.0 ± 6.3	56.5 ± 4.5

*SD* Standard deviation, *N/A* Not available, *MILS* Minimally invasive liver surgery, *OLS* Open liver surgery

#### Critical appraisal

See Table 3 for the risk of bias per study. No high level of bias was observed in any of the included articles. Risk of bias of included studies ranged from low to moderate with five studies being reported as moderate level of bias [36, 43, 46, 47, 60]. Most authors did not describe how they selected patients for MILS, resulting in selection bias. Additionally, some studies reported > 10% conversions from MILS to OLS, due to unexpected complications or other difficulties, which also introduced bias [16, 33, 37, 38, 40, 42, 43, 50, 54]. Two studies reported converted patients in their respected operative groups, according to an intention to treat analysis. Furthermore, the retrospective design of the included studies increased the risk of loss to follow-up, although this has been corrected for by including these cases in the long-term results by most studies. Twenty-one studies were propensity score matched studies, which reduces the risk of selection bias and confounding. [6, 32, 34, 36–38, 40, 42, 45, 48, 50, 53, 54] Two studies included other tumours than IHC which caused a confounding effect, which was most prevalent in the study of Cipriani et al*.*, 2019 because of the large amount of other tumours [16, 54]. Four studies had missing data, although in three studies this was limited to some pre-operative data and in the other study a statistical analysis was done to compensate for this [16, 32, 33, 50].

**Table 3:** Quality assessment of included studies

Study	Selection (max 4)	Comparability (max 2)	Outcome / Exposure (max 3)	Total Score (max 9)
Ratti et al., 2015	3	2	3	8
Uy et al., 2015	3	1	2	6
Wei et al., 2016	3	1	2	6
Lee et al., 2016	3	1	2	6
Xu et al., 2016	3	1	2	6
Ratti et al., 2018	4	2	3	9
Cipriani et al., 2019	4	2	2	8
Zhang et al., 2019	3	1	2	6
Zhu et al., 2019	4	2	3	9
Kang et al., 2020	4	2	2	8
Ratti et al., 2020 a	3	2	2	7
Ratti et al., 2020 b	4	2	3	9
Wu et al., 2020	4	1	2	7
Haber et al., 2020	3	1	2	6
Efanov et al., 2020	3	1	2	6
Ratti et al., 2021	4	2	3	9
Hobeika et al., 2021	4	2	2	8
Salehi et al., 2021	4	2	2	8
Ma et al., 2021	4	2	2	8
Chen et al. 2021	3	1	3	7
Dou et al. 2021	3	1	3	7
Jinhuan et al., 2022	4	2	3	9
Wang et al., 2022	4	2	3	9
Sucandy et al., 2022	4	2	2	8
Shapera et al., 2022	3	1	3	7
Liu et al., 2023	3	1	2	6
Wang et al., 2023	4	2	2	8
Huang et al., 2023	4	2	2	8
He et al., 2022	4	2	3	9
Brustia et al., 2022	4	2	2	8
Ma et al., 2022	4	2	2	8
Efanov et al., 2022	4	2	2	8
Pery et al., 2023	4	1	2	7
Qin et al., 2023	4	2	3	9
Xiong et al., 2023	3	2	3	8
Shen et al., 2023	4	2	3	9
Sahakyan et al., 2023	4	2	3	9

### Meta-analysis

#### Primary outcomes

**Fig. 2:**
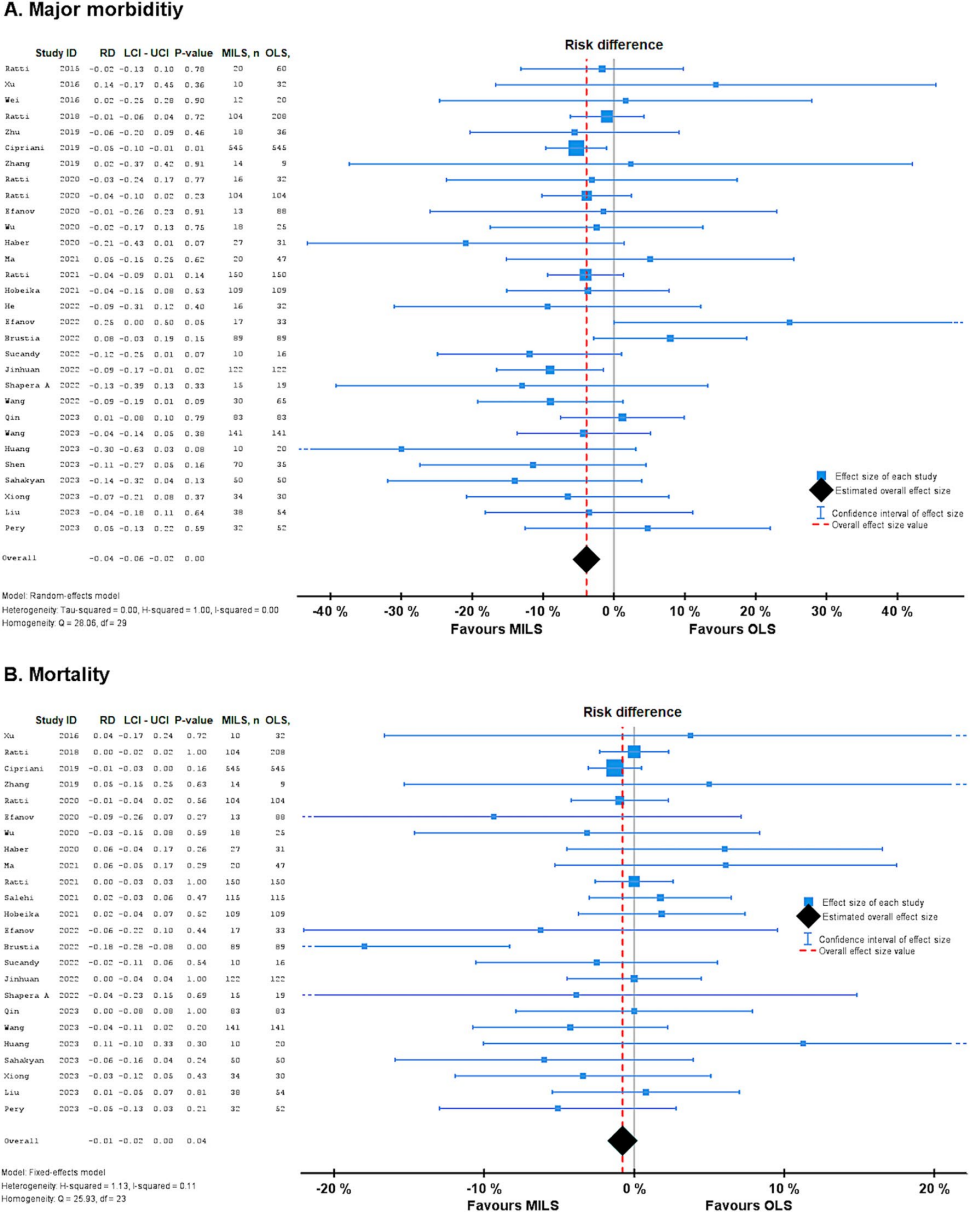
Forest plots for primary outcomes major morbidity and mortality. **A**: Forest plot of MILS versus OLS for major morbidity; **B**: Forest plot of MILS versus OLS for mortality

In 21 studies there was no difference in the overall morbidity of MILS compared to OLS, 30.8% vs 38.5% (OR 0.65, 0.53 – 0.80 P < 0.001, I^2^ = 0.97). Overall, 28 studies demonstrated significantly lower major morbidity in MILS compared to OLS, 13.3% vs 18.8% (OR 0.75, 0.62 – 0.90, P < 0.001, I^2^ = 0.97). 90-day Mortality, reported by 22 studies, was lower in MILS compared to OLS, 3.0% vs 4.5% (OR 0.69, 0.48 – 0.99, P = 0.040, I^2^ < 0.01). For more details, see Fig. 2.

#### Secondary outcomes

**Fig. 3:**
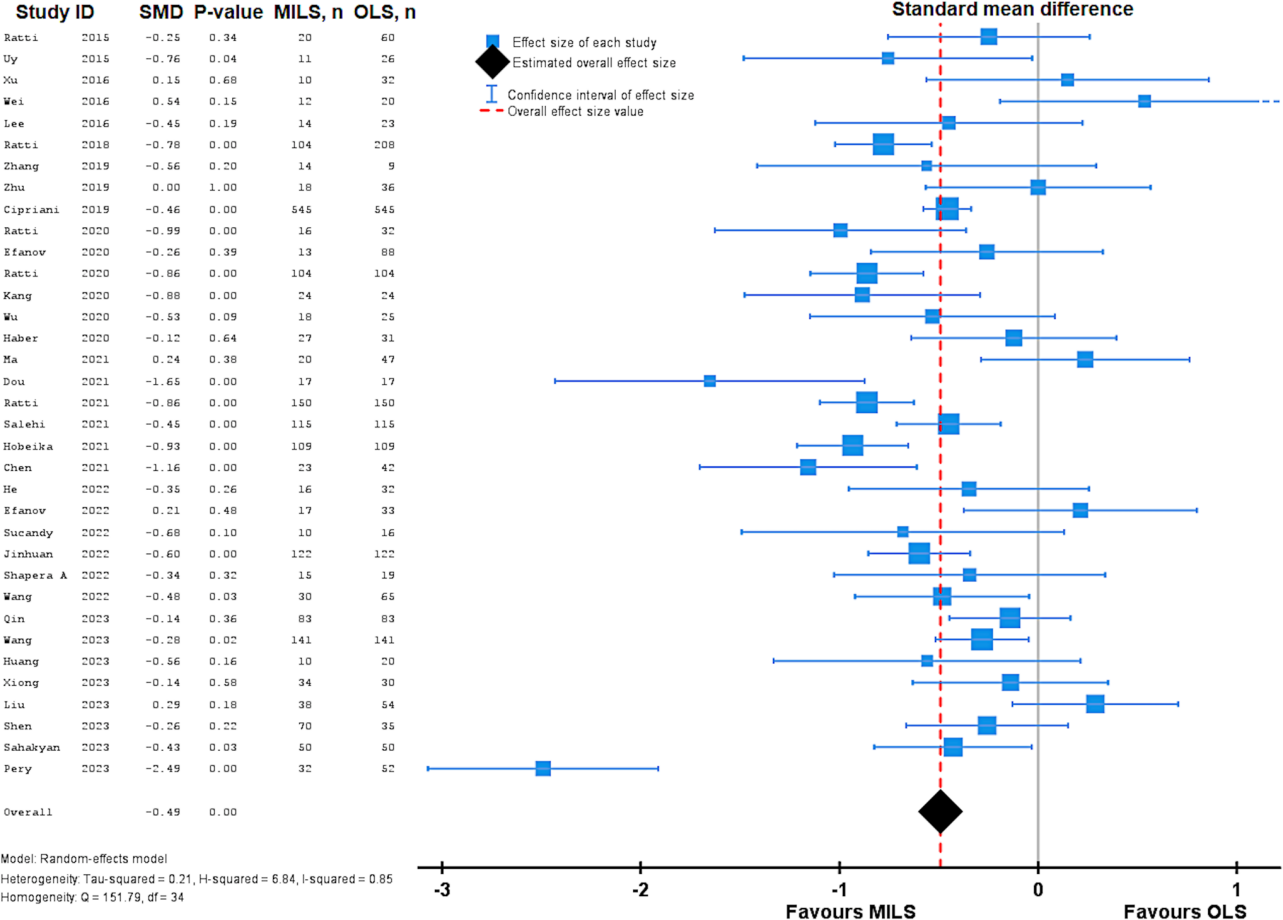
Forest plot demonstrating meta-analysis of hospital stay

Operation time, reported in 28 studies, was comparable between the minimally invasive- and open approach with respective means of 354 min vs 308 min (MD 38 min, 95%CI 13 – 63, P < 0.001, I^2^ = 0.97). Blood loss in MILS was lower compared to OLS: median 300 mL vs 350 mL (25 studies, MD 117, 95%CI − 164 – − 71, P < 0.001, I^2^ = 0.94). Hospital stay was significantly shorter in the MILS group median 6 vs 9 days (30 studies, MD − 2.14, 95%CI − 2.81 – − 1.47, P < 0.001, I^2^ = 0.85). See Fig. 3 for more details on hospital stay. Wound infection was significantly lower in MILS compared to OLS, 2.2% vs 4.8%, respectively (16 studies, OR 0.49, 0.30 – 0.80, P = 0.004, I^2^ < 0.01).

#### Other outcomes

The rate of readmission did not differ between MILS and OLS: 9.9% vs 14.1% (10 studies, OR 0.74, 95%CI 0.49 – 1.21, P = 0.158, I^2^ = 0.03). The rate of R0 resections was higher after MILS versus OLS 90.4% versus 81.4% (27 studies, OR 1.40, 95%CI 1.13 – 1.74, P = 0.002, I^2^ < 0.01). The number of lymph nodes resected did not differ significantly between MILS and OLS: median 8 vs 7 nodes (18 studies, MD 0.48, 95%CI − 0.11 – 1.06, P = 0.113, I^2^ = 0.90). The meta-analysis showed a similar rate of recurrence after a mean follow-up of 34 months (range 12–60): 62.8% versus 63.1% (19 studies, OR 1.02, 95%CI 0.99 – 1.06, P = 0.209, I^2^ < 0.01). In time-to-event meta-analysis, the DFS was significantly better in MILS vs OLS at 1-year, 3-year, and- 5-year: 71.2% vs 65.4%, HR 2.9, 95%CI 2.8 – 2.9; 49.9% vs 38.5%, HR 3.2, 95%CI 3.1 – 3.3; 37.8% vs 22.5% HR 2.7, 95%CI 2.5 – 3.0, respectively.

### Subgroup analysis

For IHC, there were significantly more minor resections (36.7% vs 34.7%, OR 1.51, 95%CI 1.08 – 2.11, P = 0.016, I^2^ = 0.74), and smaller tumour sizes (47 vs 52 mm, MD − 5.3, 95%CI − 7.5 – 3.1, P = 0.007, I^2^ = 0.46) in the MILS group versus the OLS group. Other baseline variables did not differ significantly.

Outcomes for PHC were reported by seven studies [44, 47, 48, 55, 59, 60, 65]. Overall morbidity was similar for MILS and OLS (P = 0.811), as was major morbidity (20.2% vs 29.5%, OR 0.91, 95%CI 0.64 – 1.29, P = 0.583, I^2^ < 0.01). The rate of mortality was similar between MILS versus OLS (6.4% vs 7.7%, OR 0.81, 95%CI 0.46 – 1.42, P = 0.460, I^2^ < 0.01). Operation time was significantly longer with MILS (MD 85 min, 95%CI 47 – 123, P < 0.001, I^2^ = 0.89). Length of hospital stay was significantly shorter after MILS (MD − 1.5, 95%CI − 2.1 – 0.9 P < 0.001, I^2^ = 0.58), and blood loss did not differ significantly (MD −54, 95%CI − 133 – 25, P = 0.182, I^2^ = 0.71).

Outcomes for IHC were reported by 22 studies [16, 32–34, 36–43, 49, 51–54, 57, 58, 61, 64, 67]. Overall morbidity was significantly lower for MILS versus OLS, respectively (27.4% vs 35.4%, OR 0.61, 95%CI 0.50 – 0.76, P < 0.001, I^2^ = 0.41), as was major morbidity (11.5% vs 15.1%, OR 0.65, 95%CI 0.53 – 0.81, P < 0.001, I^2^ < 0.01). Mortality was similar between groups, with MILS versus OLS being, respectively, 2.3% vs 3.5% (OR 0.65, 95%CI 0.50 – 0.76, P = 0.009, I^2^ < 0.01). Operation time did not differ significantly between MILS and OLS (MD + 10 min, 95%CI − 7 – 26, P = 0.166, I^2^ = 0.94). Blood loss and length of hospital stay were significantly lower for MILS, respectively (MD − 123 mL, 95%CI − 214 – − 122, P < 0.001, I^2^ = 0.82; and MD − 1.9 days, − 2.1 – − 1.7, P < 0.001, I^2^ = 0.87).

Meta-analysis of the studies with a robotic approach showed the following outcomes compared to that of the laparoscopic approach: morbidity (60.0% vs 30.4%), major morbidity (42.0% vs 12.2%), mortality (7.0% vs 2.8%), conversion (8.5% vs 11.2%), operative time (547 vs 292 min), blood loss (434 vs 306 mL), length of hospital stay (14.3 vs 7.7 days), HJ leaks (2.0% vs 7.0%), and wound complications (0.0% vs 2.3%) [44, 46, 49, 51, 55, 62]. Statistical power was insufficient for a valid analysis for differences, let alone for hand-assisted approach.

In 21 studies that were propensity score matched, there was a significant difference in the overall morbidity of MILS compared to OLS, 28.0% vs 36.0% (OR 0.65, P < 0.001, I^2^ = 0.08) [16, 32, 34, 35, 37, 38, 40, 42, 45, 48, 50, 51, 53–62, 66, 67]. Overall, 19 studies demonstrated significantly lower major morbidity in MILS compared to OLS: 12.6% vs 16.4% (OR 0.70, 95%CI 0.58 – 0.85, P < 0.001, I^2^ = 0.06). Furthermore, MILS was associated with significantly lower mortality in comparison to OLS: 3.0% vs 5.5%, respectively (OR 0.67, 95%CI 0.47 – 0.97, P = 0.034, I^2^ < 0.01). Finally, tumour size was significantly larger in the MILS vs OLS group, median 44 vs 43 mm, MD − 2 mm, 95%CI − 3.5 – − 0.4, P = 0.011, I^2^ = 0.38. However, R0 resection rates were higher in the MILS vs OLS group, 89.3% vs 86.9%, OR 1.36, 95%CI 1.09 – 1.71, P = 0.007, I^2^ < 0.01. In the studies without propensity score matching, morbidity, major morbidity, and mortality were similar: OR 0.51, 95%CI 0.25 – 1.03, P = 0.060, I^2^ = 0.11; OR 0.78, 95%CI 0.31 – 1.97, P = 0.597, I^2^ < 0.01; OR 0.78, 95%CI 0.50 – 1.22, P = 0.278, I^2^ < 0.01, respectively. Furthermore, R0 Resection rates were similar in the MILS vs OLS group, OR 1.86, 95%CI 0.89 – 3.92, P = 0.100, I^2^ < 0.01. Disease-free survival remained consistent between studies with- and without propensity score matching and significantly favoured the MILS group vs the OLS group.

A subgroup analysis of major vs minor liver resection was also performed for operation time. This showed a significantly longer operation time for MILS when a higher proportion of minor resections was performed. When a higher proportion of major resections was performed the difference in operative time was non-significant.

Nineteen studies, of which 12 focused on PHC, reported on biliodigestive anastomoses in their cohort comprising 1180 patients of which 498 laparoscopic, 65 robotic, and 617 open [33, 35, 41, 44–50, 53, 55–58, 60, 62, 65, 66]. In this group, morbidity, major morbidity, and mortality were similar between MILS and OLS. The rate of biliary leakage was comparable between MILS and OLS: 12.1% versus 11.9% (OR 0.97, 95%CI 0.59 – 1.58, P = 0.897, I^2^ < 0.01).

### Sensitivity analyses

Sensitivity analysis focused on study quality by isolating the 11 studies deemed to be of the highest methodological quality based on the Newcastle–Ottawa Scale (NOS) (maximum attainable score of 9 points), others were excluded (< 9 points). There was a significant difference in the overall morbidity of MILS compared to OLS, 15.6% vs 24.1% (OR 0.54, 95%CI 0.52 – 0.57, P < 0.001, I^2^ < 0.01) [37, 42, 53, 54]. Overall, eight studies demonstrated significantly lower major morbidity in MILS compared to OLS: 7.2% vs 9.4% (OR 0.43, 95%CI 0.41 – 0.46, P < 0.001, I^2^ = 0.63) [35, 37, 42, 50, 53, 54, 56, 61]. Furthermore, MILS had significantly lower mortality in comparison to OLS: 2.7% vs 4.5%, respectively (OR 0.40, 95%CI 0.37 – 0.42, P < 0.001, I^2^ = 0.93) [35, 37, 42, 50, 53, 54, 61].

Operative time was significantly longer in the MILS vs OLS group, median 270 min vs 230 min, MD + 35 min, 95%CI 26 – 43, P < 0.001, I^2^ = 0.47.[35, 37, 42, 53, 54, 56, 61] Blood loss was significantly lower in the MILS vs OLS group, median 200 mL vs 350 mL, MD −159 mL, 95%CI − 233 – − 0.85, P < 0.001, I^2^ = 0.91.[35, 37, 42, 53, 54, 56] Hospital stay was significantly shorter in the MILS vs OLS group, median 4 days vs 6 days, MD − 0.56 days, 95%CI − 0.81 – − 0.31, P < 0.001, I^2^ = 0.77 [35, 37, 42, 50, 53, 54, 56, 61].

Finally, tumour size was significantly smaller in the MILS vs OLS group, mean 42 vs 48 mm, MD − 2.9 mm, 95%CI − 4.6 – − 1.3, P < 0.001, I^2^ = 0.38. However, number lymph nodes resected were higher in the MILS vs OLS group, median 8 vs 7 nodes, MD 1.06, 95%CI 0.45 – 1.67, P < 0.001, I^2^ < 0.89 [35, 37, 42, 54, 56]. Moreover, R0 resection rates were higher in the MILS vs OLS group, 94.9% vs 91.7%, OR 1.03, 95%CI 1.00 – 1.05, P = 0.040, I^2^ < 0.01.[35, 37, 42, 50, 53, 54, 56] Recurrence rates [37, 42, 50, 53, 54, 56] and disease-free survival rates[37, 42, 54] were not significantly different.

Based on the study period and cases included, a higher annual volume of MILS correlated with a significantly lower morbidity rate, major morbidity rate and mortality rate (Rho = 0.82, P < 0.001; Rho = 0.74, P < 0.001; Rho = 0.82, P < 0.001, respectively).

A higher conversion rate correlated with a higher morbidity rate (Rho = 0.24, P < 0.001), a higher rate of major morbidity (Rho = 0.47, P < 0.001), and a higher rate of mortality (Rho = 0.37, P < 0.001).

## Discussion

This first systematic review of comparative studies on the use of MILS for PHC and IHC including 37 studies and 4863 patients finds that, in highly selected patients, MILS may result in lower rates of major morbidity and mortality as compared to OLS. MILS may result in significantly shorter length of stay with comparable operative time. The sensitivity analysis shows more favourable outcomes for MILS in higher volume centres and after propensity score matching. Results may show no significant differences between PHC and IHC in the impact of MILS compared to OLS on the primary outcomes.

**Fig. 4:**
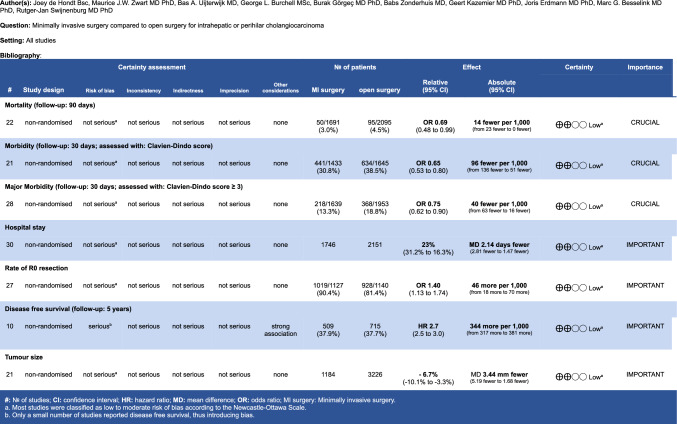
GRADE evidence table

**Fig. 5:**
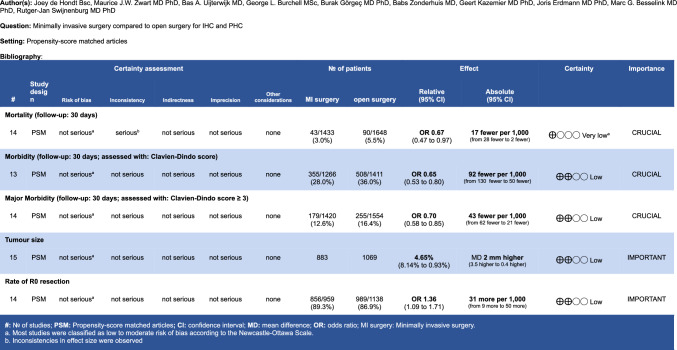
GRADE evidence table PSM articles

The overall quality of evidence in this systematic review was low, primarily because the crucial outcomes in the GRADE evidence table (Fig. 4) were of low quality, highlighting the need for randomised controlled trials (RCTs). Despite this, the results showed considerable consistency, and the effect size in the analysis of propensity score matched (PSM) articles increased when confounding factors were eliminated. However, this review did not include any RCTs. A comparison of the 95% confidence intervals for primary outcomes between PSM and non-PSM articles reveals that non-PSM articles have a significantly wider and non-significant interval, suggesting potential bias in these non-PSM studies. When the quality of evidence for the PSM articles was assessed using GRADE (Fig. 5), it was also found to be low overall. Furthermore, inconsistent results for mortality in the PSM-matched articles led to a downgrading of this evidence to very low. Nevertheless, the observed effects remained consistent, and in some cases larger, when the sensitivity analysis was restricted to only the highest-quality articles. This consistency supports the robustness of the main findings and suggests that potential bias in the broader dataset might have underestimated the true effect.

The current systematic review is the first to include both IHC and PHC. A 2021 meta-analysis by Patrone et al. included nine studies and 3012 patients, of which 562 underwent MIS.[69] The current study includes 37 studies, thus being larger. Furthermore, the present study was the first meta-analysis including only comparative studies for both IHC and PHC, thereby aiming to limit selection bias. For more details, see Appendix3.

Analysis of secondary outcomes shows that MILS may result in a large reduction of blood loss, shorter post-operative hospital stay and lower occurrence of wound infection. These results resonate with the general inherent benefits of minimally invasive surgery [9–15]. Although a longer operative time was expected as an inherent downside of MILS, this study only finds a slight increase (14 min longer) in operative time. However, when a higher proportion of minor resections is performed there may be a significantly longer operative time for MILS, possibly due to more difficult laparoscopic resections with a larger area of resection or a larger portion of time lost on docking and setting up the laparoscopic equipment [9–15]. The similar operation time may be explained by the large experience of the surgeons in some studies [37, 38, 42, 54]. The difference between hypothesis and results in operative time may also be an indication of publication bias or limitations of this study. The other outcomes were split in surgical and oncological outcomes. The surgical outcomes may show no significant differences in the extent of resection or readmission rate. The similar extent of resection between MILS and OLS may be an indication of some pre-operative similarities between the two operative groups (MILS and OLS) due to propensity score matching and thus less confounding in this study.

Impaired oncological outcomes are one of the main concerns in the use of MILS for IHC [9–15]. In this study, an examination of the oncological outcomes may suggest that MILS was non-inferior or sometimes superior to OLS. The oncological outcomes may suggest MILS performs significantly better in regards to DFS. The recurrence rate may be similar, with significantly higher rates of negative resection margins in MILS, as compared to OLS. It should be taken into account that tumour size may be significantly smaller in the MILS group, which may explain the higher rates of negative margins. However in the analysis of PSM-matched articles tumour size may be actually larger in the MILS group, whilst negative margins remain significantly higher in the MILS group. The number of resected lymph nodes is similar between groups, although as Ratti et al*.*(2018) mentioned in his study, this outcome is of limited value due to the AJCC advising the resection of six lymph nodes for IHC [39, 54]. Indeed, both MILS and OLS show a comparable lymph node yield above six [39, 54]. Therefore, this study may suggests that MILS is an oncological viable option for highly selected cholangiocarcinomas. Furthermore, Song et al. demonstrated that MILS causes less liver function damage, a better inflammatory response, both contributing to a faster recovery, and similar oncological outcomes compared to OLS [70].

The results of this study should be interpreted in light of several limitations. First, all studies but one, are of a retrospective design. Therefore, there is a demand for registries, prospective and randomised studies to increase the level of evidence on the topic. Although the absence of randomised studies results in a higher risk of bias and confounding (as mentioned in the critical appraisal), there are no studies with high levels of bias. In addition, most studies performed a propensity score matched analysis, which limits bias. Second, there were differences in the applied technique, *i.e.* only four studies reported whether laparoscopic surgery was purely laparoscopic or employed hand-assisted surgery, which limited the subgroup analysis [43]. Additionally, only 75 robotic procedures are reported as of now, thus more research on the robotic technique is required. Third, further risk of bias may be present for oncological outcomes due to the significantly smaller tumour size in patients undergoing MILS, making the results less generalizable. However, in the analysis of PSM articles tumour size is significantly larger, whilst the rate of negative margins remains significantly higher in the MILS group. Fourth, a small number of studies reported DFS and readmission rate, and the location of recurrence (local or distant). Fifth, only a small number of articles report results on PHC, thus making this a comparatively small cohort compared to IHC. As PHC prevalence is higher in Asian countries, there are numerous reports in Chinese that had to be translated for the current meta-analysis [71]. Finally, whilst we recognise the potential significance of vascular involvement, only six of the included studies reported data on this aspect, each with fewer than ten cases. Given the limited number of cases and the infrequency of reporting across the studies, we determine that a focused analysis of vascular involvement would likely not yield meaningful insights or substantially alter the conclusions of our systematic review. However, we acknowledge that this is an area that may warrant further investigation in future research with a larger sample size and more consistent reporting.

Robotic surgery poses a great platform for innovations in new surgical techniques and imaging for the future, as shown by Procopio et al.[72] However, its application in cholangiocarcinoma treatment remains limited, with only 75 cases reported in literature to date. Current studies suggest possible comparable outcomes between robotic and laparoscopic approaches, but sample sizes are too small for a valid statistical analysis and definitive conclusions. Further research is needed to explore the potential of robotic procedures, particularly for intrahepatic cholangiocarcinoma (IHC) and perihilar cholangiocarcinoma (PHC).

In conclusion, this systematic review and meta-analysis may suggest that in selected patients, and in patients after propensity score matching, MILS may result in an acceptable safety profile with potential benefits in patient recovery and some oncological outcomes as compared to OLS. Benefits of MILS for patients with IHC and PHC needs to be confirmed in future randomised trials investigating MILS, preferably robot-assisted surgery, versus OLS in high volume centres with a significantly long follow-up to adequately analyse oncological outcomes.

## Appendix 1: Detailed systematic search

### PUBMED

#### Minimally invasive liver resections

Population: (“Cholangiocarcinoma”[Mesh] OR cholangiocarcinoma* [tiab] OR Klatskin[tiab] OR “Bile Duct Neoplasms”[Mesh] OR Bile Duct cancer*[tiab] OR Bile Duct neoplasm*[tiab]) AND ("Bile Ducts, Intrahepatic"[Mesh] OR intrahep*[tiab] OR perihilar[tiab] OR "Neoplasms, Cystic, Mucinous, and Serous"[Mesh] OR cystic [tiab] OR hilar [tiab]).

Intervention: AND (“Laparoscopy”[Mesh] OR laparoscop* [tiab] OR “Hand-Assisted Laparoscopy”[Mesh] OR Hand Assisted Laparoscopy [tiab] OR “Robotic Surgical Procedures”[Mesh] OR robot* [tiab] OR “Minimally Invasive Surgical Procedures”[Mesh] OR Minimally Invasive [tiab] OR hybrid [tiab]).

Comparison: AND (open [tiab] OR conventional [tiab]).

#### Minimally invasive hepatopancreatoduodenectomy

Population: ((Bile-Duct* OR intrahep* OR perihilar OR hilar-plate)(Neoplasm* OR cancer* OR tumor* OR tumour* OR carcinoma* OR adenocarcinoma*)).ti,ab,kf.

Intervention: ((hepatopancreatoduodenectom* OR hepato-pancreatoduodenectom* OR hepatopancreatico*).ti,ab,kf.) AND (Exp Laparoscopy/ OR Exp Robotic Surgical Procedures/ OR Exp Minimally Invasive Surgical Procedures/ OR (laparoscop* OR robot* OR da-vinci OR Minimally-Invasive* OR hybrid OR key-hole OR keyhole OR hand-assisted).ti,ab,kw).

Comparison: AND (open [tiab] OR conventional [tiab]).

#### Minimally invasive pancreatoduodenectomy

Population: (distal cholangiocarcinoma*[tiab] OR periampulla*[tiab] OR periampull*[tiab] OR distal bile duct[tiab] OR ampulla of vater*[tiab] OR ampullary adenocarcinoma*[tiab] OR ampul*[tiab] distal cholangiocarcinoma.

Intervention: ("Pancreaticoduodenectomy"[Mesh] OR "Pancreatectomy"[Mesh] OR pancreatoduodenectom*[tiab] OR pancreaticoduodenectom*[tiab] OR pancreato duodenectom*[tiab] OR pancreatico duodenectom*[tiab] OR pancreatic duodenectom*[tiab] OR pancreato duodenal resection*[tiab] OR whipple*[tiab]).

Comparison: ("Laparoscopy"[Mesh] OR laparoscop*[tiab] OR LDP[tiab] OR "Robotic Surgical Procedures"[Mesh] OR "Robotics"[Mesh] OR robot*[tiab] OR RDP[tiab]) open pancreatoduodenectomy.

#### EMBASE—detailed search

- 1. ‘bile duct carcinoma’/exp OR ‘hepatobiliary system cancer’/exp OR ‘biliary tract tumor’/exp OR (Cholangiocarcinoma* OR Cholangiocellular-Carcinoma* OR Klatskin*):ti,ab,kw OR ( (Bile-Duct* OR intrahep* OR perihilar OR hilar-plate) NEAR/5(Neoplasm* OR cancer* OR tumor* OR tumour* OR carcinoma* OR adenocarcinoma*)):ti,ab,kw OR (hemihepatectom* OR segmentectom* OR hepatopancreatoduodenectom*):ti,ab,kw → 412,918.
- 2. ‘laparoscopy’/exp OR ‘robot assisted surgery’/exp OR ‘minimally invasive surgery’/exp OR (laparoscop* OR robot* OR da-vinci OR Minimally-Invasive* OR hybrid OR key-hole OR keyhole OR hand-assisted):ti,ab,kw → 760,504.
- 3. (open OR conventional OR laparotom*):ti,ab,kw → 1,736,069.
- 4. #1 AND #2 AND #3 → 5,308.
- 5. #4 AND (‘article’/it OR ‘article in press’/it OR ‘review’/it) → 3114.

#### COCHRANE—detailed search

- 1. (Cholangiocarcinoma* OR Cholangiocellular-Carcinoma* OR Klatskin* OR ( (Bile-Duct* OR intrahep* OR perihilar OR hilar-plate) AND (Neoplasm* OR cancer* OR tumor* OR tumour* OR carcinoma* OR adenocarcinoma*)) OR hemihepatectom* OR segmentectom* OR hepatopancreatoduodenectom*):ti,ab,kw → 2,966.
- 2. (laparoscop* OR robot* OR da-vinci OR Minimally-Invasive* OR hybrid OR key-hole OR keyhole OR hand-assisted):ti,ab,kw → 46,712.
- 3. (open OR conventional OR laparotom*):ti,ab,kw → 222,713.
- 4. #1 AND #2 AND #3 → 101.

## Appendix 3. Table comparing the current meta-analysis to systematic reviews with a similar scope.

**Table:**

Authors	Period (up to) # Patients # Reports	Procedure	Studies included	Location	Conclusions	Limitations
de Hondt, Zwart 2024	June 2024 N = 3511 R = 37	75 Robotic 2106 Lap 1944 Open	comparative (PSM matched)	All	*“The superior mortality and superior morbidity … minimally invasive surgery is safe and effective in … intrahepatic- and perihilar cholangiocarcinoma. … minimally invasive surgery can be safely recommended …, especially in higher volume centres.”*	*“… absence of randomised studies … no studies with critical level of bias. … most studies performed a propensity score matched analysis, which limited bias.”*
Wu [73] 2024	Jan 2024 N = 513 R = 5	231 Robotic 282 Open	Comparative		*“robotic-assisted surgery for CCA offers significant advantages … intraoperative blood loss, lymph node harvest, and length of hospital stay, demonstrating its feasibility and safety. However, further confirmation of its long-term effects and oncological outcomes is required.”*	*“all literature was retrospective. The small sample size attenuated the credibility of the results. (3) The collected data were insufficient to adequately assess the prognosis and patient survival could not be collected. The results of operative time and POCs exhibit high heterogeneity.”*
Li [71] 2023	April 2023 N = 1054 R = 6	518 Lap 536 Open	PSM matched only	IH	*“LH has better surgical outcomes and comparable oncological outcomes and survival outcomes “... Therefore, laparoscopy is at least not inferior to open surgery for intrahepatic cholangiocarcinoma*	*“… all retrospective studies and lacked RCTs and other prospective studies.”*
Aliseda [26] 2023	May 2022 N = 1042 R = 6	512 Lap 530 Open	PSM matched only	IH	*“…support laparoscopic surgery in iCC. LLR was associated with a significantly longer OS. …in well-selected patients, laparoscopic surgery offers OS outcomes at least equivalent to those of OLR”*	*“may be unmeasured confounding variables. …the era effect, presence of heterogeneity in some analyses and publication bias need to be considered.”*
Berardi [74] 2023	Nov 2022 N = 372 R = 18	310 Lap 62 Robotic	Non-comparative	Hilar	*“Minimally invasive surgery for patients with perihilar cholangiocarcinoma is feasible …good perioperative outcomes and safe oncological results, both laparoscopic and robotic. MIS must be performed in high-volume units … expert minimally invasive surgeons”*	*“the low quality of the studies, all retrospective and only a few being comparative or case-controlled, with few patients included. …almost 80% of the articles were from China.”*
Brolese [75] 2022	May 2022 N = 109 R = 12	109 Robotic	Case series	Hilar	*“this systematic review was inconclusive … RS for the treatment of HC could certainly represent the best tool for future meticulous and precise surgery.”*	*“The quality of the included studies … low or very low. Due to the absence of a control group unable to conduct comparative meta-analysis. …patients may have been clinically selected patients.”*
Patron [69] 2021	January 2021 N = 3012 R = 9	562 MIS 2450 Open	Comparative	IH	*“Minimally invasive resection of ICC has some short-term benefits and it is safe and feasible only in selected centers with a high experience in laparoscopic approach for liver surgery.”*	*“… tumor with a diameter* < *5 cm, … no vascular or biliary reconstructions were planned. Further studies are needed … long term oncologic outcomes*
Tang [17] 2021	July 2020 N = 382 R = 9	164 MIS 218 Open	Comparative	Hilar	*“The safety and feasibility of MIS for HCCA is acceptable in selected patients. … providing comparable outcomes associated with a benefit of minimal invasiveness and its application should be considered more.”*	*First, … small retrospective cohort studies, no high volume study or well-designed randomized controlled trial was included*
Cipriani [18] 2021	July 2020 N = 193 R = 19	92 Lap 101 Robotic	non-comparative	Hilar	*“In selected patients, minimally-invasive surgery for Klatskin tumors appears feasible, safe, satisfactory for perioperative outcomes and adequate for oncologic results.”*	*“However, … few studies, limited in patient numbers and with allocation criteria more restrictive than open, … non-comparative design: evidence of higher quality is recommended.”*
Machairas [19] 2021	June 2020 N = 2872 R = 8	552 Lap 2320 Open	Comparative	IH	*“LLR seems to benefit patients with iCCA in terms of short-term outcomes, whilst long-term outcomes are comparable among the two approaches.”*	*“Moreover, there was a notable heterogeneity, … selection and attrition bias as well as selective reporting. … interstudy variability ….”*
Rahnemai-Azar [20] 2020	January 2020 N = 30 R = 8	30	Non-comparative	Hilar	*“MIS may be a safe and feasible approach at high-volume centers with robust expertise in the management of patients with pCCA.”*	*“Further studies with larger number of patients are required prior to universal application of MIS for pCCA.”*
Wang [21] 2021	April 2020 N = 205 R = 23	101 Robot 99 Lap	Non-comparative	Hilar	*“With innovations in technology and gradual accumulation of surgical experience, the feasibility and safety of performing Lap and Rob for HC will improve.”*	*“With innovations in technology and gradual accumulation of surgical experience, the feasibility and safety of performing Lap and Rob for HC will improve.”*
Guerrini [22] 2020	October 2019 N = 204 R = 4	75 Lap 129 Open	Comparitive	IH	*“Laparoscopic liver resection for ICC … better surgical outcomes, … short-term benefits without negatively affecting oncologic adequacy …, However, a higher LND rate was observed in the open group.”*	*Due to the risk of bias and the statistical heterogeneity between the studies …, further RCTs are needed to reach stronger scientific conclusions*
Shiraiwa [23] 2020	July 2019 N = 323 R = 25	10 Robot 152 Lap 161 Open	5 comparitive + 20 non-comparitive	Hilar and IH	*“…. Regarding iCCA, LLR had lower blood loss and less … Pringle maneuver. … (OLR) were performed more for major hepatectomies, with better lymphadenectomy rates and higher number of harvested lymph nodes. …”*	*“…. High heterogeneity and selection bias were suggested for iCCA studies.”*
Franken [12] 2019	September 2017 N = 174 R = 21	59 Robot 82 Lap 1 Hybrid 32 Open	1 comparitive + 20 non-comparitive	Hilar	*“…. Significant increased operative time and higher morbidity rate with MIS. … “ (more studies should be done…)*	*“…. The available evidence on MIS for PHC is limited and generally of poor quality. …”*

*IH* Intrahepatic
